# Breaking the barrier: Nanoparticle-enhanced radiotherapy as the new vanguard in brain tumor treatment

**DOI:** 10.3389/fphar.2024.1394816

**Published:** 2024-07-03

**Authors:** Shi feng Liu, Meng Jiao Li, Bing Liang, Wenshe Sun, Yingchun Shao, Xiaokun Hu, Dongming Xing

**Affiliations:** ^1^ The Affiliated Hospital of Qingdao University, Qingdao, China; ^2^ Qingdao Cancer Institute, Qingdao University, Qingdao, China

**Keywords:** nanoparticle-enhanced radiotherapy, brain tumor, blood-brain barrier, radio sensitization, clinical translation, regulatory landscapes

## Abstract

The pursuit of effective treatments for brain tumors has increasingly focused on the promising area of nanoparticle-enhanced radiotherapy (NERT). This review elucidates the context and significance of NERT, with a particular emphasis on its application in brain tumor therapy—a field where traditional treatments often encounter obstacles due to the blood-brain barrier (BBB) and tumor cells’ inherent resistance. The aims of this review include synthesizing recent advancements, analyzing action mechanisms, and assessing the clinical potential and challenges associated with nanoparticle (NP) use in radiotherapy enhancement. Preliminary preclinical studies have established a foundation for NERT, demonstrating that nanoparticles (NPs) can serve as radiosensitizers, thereby intensifying radiotherapy’s efficacy. Investigations into various NP types, such as metallic, magnetic, and polymeric, have each unveiled distinct interactions with ionizing radiation, leading to an augmented destruction of tumor cells. These interactions, encompassing physical dose enhancement and biological and chemical radio sensitization, are crucial to the NERT strategy. Although clinical studies are in their early phases, initial trials have shown promising results in terms of tumor response rates and survival, albeit with mindful consideration of toxicity profiles. This review examines pivotal studies affirming NERT’s efficacy and safety. NPs have the potential to revolutionize radiotherapy by overcoming challenges in targeted delivery, reducing off-target effects, and harmonizing with other modalities. Future directions include refining NP formulations, personalizing therapies, and navigating regulatory pathways. NERT holds promise to transform brain tumor treatment and provide hope for patients.

## 1 Introduction

### 1.1 Background on brain tumors

Brain tumors encompass a diverse array of neoplasms, each with unique pathological and clinical characteristics. The classification of brain tumors is primarily based on the cell of origin, as well as the molecular and genetic profile, which provides insight into the tumor’s behavior and potential responsiveness to treatment (Louis, 2016). Brain tumors encompass a diverse array of neoplasms, each with unique pathological and clinical characteristics.

Gliomas, which originate from glial cells, are the most common primary brain tumors in adults. They are further classified into subtypes based on their histological features and molecular profiles, with glioblastoma (GBM) being the most aggressive and prevalent subtype (Schueller et al., 2005; Jakacki et al., 2016). GBM is characterized by rapid proliferation, diffuse infiltration, and a high degree of intratumoral heterogeneity, contributing to its poor prognosis (Drean et al., 2016).

Meningiomas, which arise from the meninges surrounding the brain, are the second most common primary brain tumors in adults. Although most meningiomas are benign (WHO grade I), a subset exhibits atypical (WHO grade II) or malignant (WHO grade III) features, associated with higher recurrence rates and poorer outcomes (Ding et al., 2013; Sahm et al., 2017).

Other brain tumor types in which NERT has been explored include primitive neuroectodermal tumors (PNETs), such as medulloblastoma (Fink et al., 2015), which is the most common malignant brain tumor in children, and brain metastases, which are secondary tumors originating from primary cancers elsewhere in the body (Chen X. et al., 2023).

Treatment strategies for brain tumors are tailored according to the tumor type, size, location, and patient’s overall health status (Qiu et al., 2022). Surgical resection remains the cornerstone of treatment for accessible brain tumors, aiming to maximize tumor removal while preserving neurological function. However, the infiltrative nature of gliomas often precludes complete resection. Adjuvant therapies, including radiotherapy and chemotherapy, are critical components of the treatment regimen, especially for high-grade tumors. Temozolomide, in combination with radiotherapy, has been shown to improve survival in patients with GBM (Stupp et al., 2005). Despite these interventions, the prognosis for malignant brain tumors remains poor, underscoring the need for more effective therapeutic approaches. Despite advancements in surgical techniques, radiation therapy, and chemotherapy, the prognosis for patients with malignant brain tumors remains poor (Corti et al., 2022). The BBB presents a significant obstacle to the effective delivery of therapeutic agents, while the inherent resistance of tumor cells to conventional treatments further complicates the management of these neoplasms (Li et al., 2022). These challenges underscore the need for innovative strategies, such as NERT, which has the potential to overcome these barriers and improve treatment outcomes.

The treatment of brain tumors is fraught with challenges, paramount among them being the BBB and tumor heterogeneity. The BBB is a formidable obstacle to the delivery of therapeutic agents, limiting the efficacy of systemic chemotherapy. NP-based drug delivery systems are being explored to circumvent the BBB and achieve targeted drug delivery (Saraiva C. et al., 2016). Tumor heterogeneity, both inter- and intra-tumoral, complicates treatment by providing a reservoir of cells with varying sensitivity to therapies, facilitating recurrence and resistance. Understanding the molecular underpinnings of this heterogeneity is essential for the development of targeted therapies and personalized medicine approaches (Patel et al., 2014). While strides have been made in the understanding and treatment of brain tumors, significant challenges remain. Advances in molecular biology and genomics have started to inform more targeted and individualized treatment strategies, offering hope for improved outcomes. Continued research is essential to overcome the current limitations and to provide patients with brain tumors a better quality of life and a more optimistic prognosis.

### 1.2 Chemotherapy in cerebral gliomas

Chemotherapy plays a crucial role in the management of cerebral gliomas, particularly in high-grade tumors such as glioblastoma. The standard of care for newly diagnosed glioblastoma involves maximal safe surgical resection followed by concurrent chemoradiotherapy with temozolomide (TMZ) and adjuvant TMZ (Yan et al., 2016). TMZ is an oral alkylating agent that induces DNA damage and cell death in tumor cells. The addition of TMZ to radiotherapy has been shown to improve overall survival and progression-free survival compared to radiotherapy alone (Lonardi et al., 2005).

However, the efficacy of chemotherapy in gliomas is often limited by the presence of the BBB, which restricts the entry of many chemotherapeutic agents into the brain. To overcome this challenge, various strategies have been explored, including the use of NPs as drug delivery vehicles. NPs can be engineered to cross the BBB and deliver chemotherapeutic agents directly to the tumor site, thereby increasing drug concentrations in the tumor while minimizing systemic toxicity (Chester et al., 2018).

Another challenge in the chemotherapy of gliomas is the development of drug resistance. Glioma cells can acquire resistance to TMZ through various mechanisms, such as the upregulation of DNA repair enzymes or the expression of drug efflux transporters (Campos et al., 2016). To address this issue, combination therapies involving multiple chemotherapeutic agents or targeted therapies have been investigated. For example, the combination of TMZ with other agents, such as lomustine or bevacizumab, has shown promise in improving outcomes in recurrent glioblastoma. Chemotherapy is an essential component of the multimodal treatment approach for cerebral gliomas. The use of NPs as drug delivery vehicles and the development of combination therapies are promising strategies to overcome the challenges associated with chemotherapy in gliomas, such as BBB and drug resistance (Liau et al., 2023). Further research is needed to optimize these approaches and improve patient outcomes.

### 1.3 Radiotherapy in brain tumor treatment

Radiotherapy remains a principal modality in the management of brain tumors, both as a primary treatment and as an adjuvant to surgery. The fundamental principle of radiotherapy lies in the delivery of ionizing radiation to induce DNA damage, thereby inhibiting tumor growth and causing cell death. Precise targeting of the radiation dose is critical to maximize tumor control while minimizing damage to surrounding normal brain tissue (Hall et al., 2021).

Technical advancements have ushered in sophisticated methods of radiotherapy delivery. Conformal techniques such as Three-dimensional conformal radiotherapy ensure the radiation beams conform to the geometrical shape of the tumor. Intensity-modulated radiotherapy further refines this approach by modulating the intensity of radiation beams, allowing for higher doses to be delivered to the tumor while sparing adjacent healthy tissue (Mayo et al., 2010).

Stereotactic radiosurgery (SRS) and stereotactic radiotherapy (SRT) are highly precise forms of radiotherapy that deliver a high dose of radiation to a small focal area, typically used for smaller brain tumors or those in critical locations. SRS is usually given in a single session, whereas SRT delivers the dose over several sessions (Leksell, 1983).

Proton beam therapy is a more recent development that uses protons rather than photons, offering a distinct advantage in the form of the Bragg peak, which allows for most of the proton’s energy to be deposited at a specific depth, minimizing exposure to surrounding tissues (Brada et al., 2009).

Despite the therapeutic benefits, conventional radiotherapy is not without limitations. The diffuse and infiltrative nature of certain brain tumors, such as high-grade gliomas, makes it challenging to define the target margins for radiotherapy. Moreover, the radiation dose that can be safely delivered is often limited by the tolerance of normal brain tissue, which can lead to potential long-term neurocognitive effects, particularly in pediatric patients (Merchant et al., 2009).

Resistance to radiotherapy is another significant challenge. The presence of hypoxic regions within the tumor has been associated with radioresistance, as oxygen is a potent radiosensitizer. Tumor cells in these hypoxic zones are less susceptible to the DNA damage caused by radiation (Moeller et al., 2007). Additionally, the inherent heterogeneity of brain tumors at the cellular and molecular levels contributes to variable responses to radiotherapy. Genetic mutations, such as those affecting the TP53 or PTEN genes, can alter the sensitivity of tumor cells to radiation (Begg et al., 2011).

The BBB presents a formidable challenge in the treatment of brain tumors, as it restricts the entry of most therapeutic agents into the brain parenchyma. The tight junctions between endothelial cells and the presence of efflux transporters limit the penetration of conventional chemotherapeutic drugs and radiotherapy sensitizers (Wu et al., 2023). NERT has emerged as a promising strategy to overcome the BBB and deliver therapeutic agents directly to the tumor site. By engineering NPs with specific surface modifications, such as ligands targeting BBB receptors or cell-penetrating peptides, researchers aim to facilitate the transport of NPs across the BBB and enhance their accumulation within brain tumors (Burger et al., 2023).

### 1.4 NPs in medicine

**FIGURE 1:**
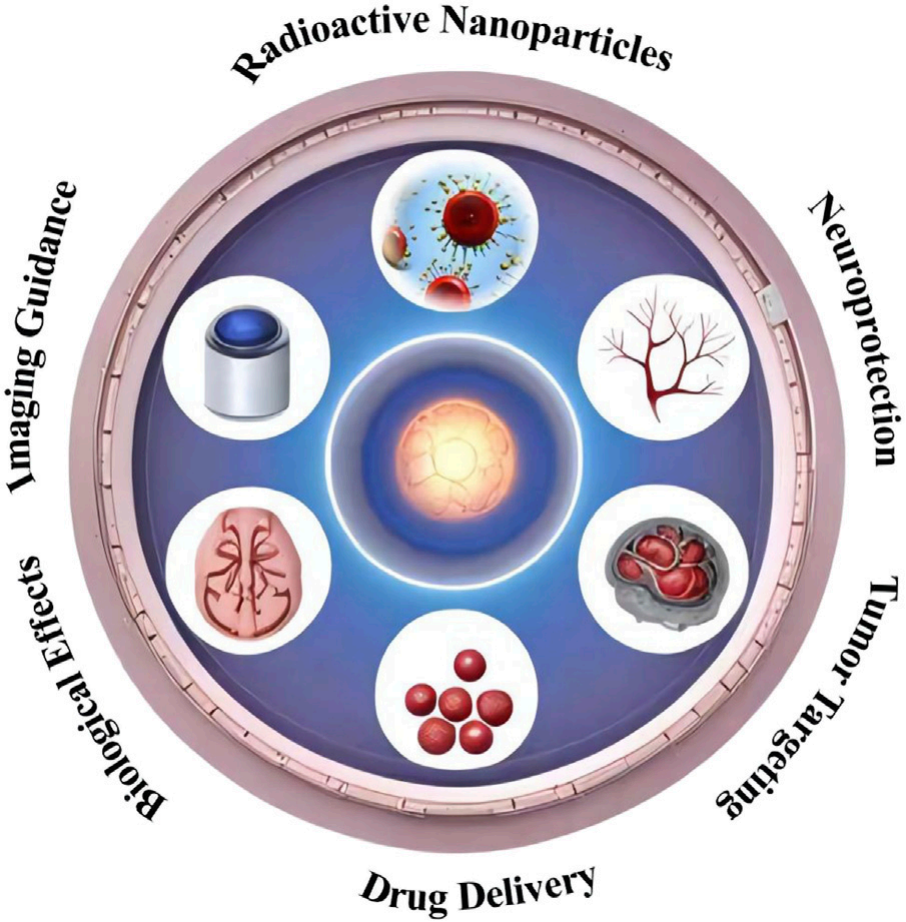
Schematic representation of the multifaceted synergistic effects of nanocarrier-enhanced radiotherapy for brain tumors. The illustration depicts the key mechanisms by which nanocarriers can enhance the efficacy of radiotherapy, including targeted delivery, radiosensitization, and the ability to overcome the blood-brain barrier.

NPs have emerged as a revolutionary technology in the field of medicine, offering novel solutions to challenges in diagnosis, treatment, and drug delivery. These particles, typically ranging from 1 to 100 nm in size, possess unique chemical, physical, and biological properties due to their nanoscale dimensions and high surface area to volume ratio. In medicine, NPs are engineered for specific applications including targeted drug delivery, imaging, and as therapeutic agents (Figure 1).

The medical applications of NPs are vast and diverse. For instance, gold NPs are utilized in photothermal therapy, where they are designed to accumulate in tumor tissues and convert absorbed light into heat, causing localized tumor cell death (Huang et al., 2008). Quantum dots, semiconductor NPs with exceptional optical properties, are employed in imaging for their stable fluorescence and ability to be tuned to emit light at various wavelengths (Medintz et al., 2008). Lipid-based NPs such as liposomes have been used for drug delivery, capitalizing on their biocompatibility and ability to encapsulate both hydrophilic and hydrophobic drugs (Sercombe et al., 2015).

The integration of NPs into medical practice is not without challenges. The interaction of NPs with biological systems raises concerns regarding toxicity, immunogenicity, and environmental impact (De Jong and Borm, 2008). The development of NPs for medical use must carefully consider these factors, ensuring safety and efficacy through rigorous preclinical and clinical evaluations.

In drug delivery, NPs offer significant advantages over conventional methods. They can be designed to improve the solubility of poorly water-soluble drugs, enhance drug stability, and control drug release rates, ensuring a sustained therapeutic effect (Mura et al., 2013). Targeted drug delivery is another key advantage; NPs can be functionalized with ligands such as antibodies or peptides to recognize and bind to specific cell types or tissues, thereby increasing the concentration of the drug at the desired site of action while minimizing systemic exposure and side effects (Peer et al., 2007).

NPs also play a pivotal role in overcoming biological barriers. For instance, polymeric NPs have been engineered to cross the BBB, a significant obstacle in the treatment of central nervous system disorders. These NPs can transport therapeutic agents across the BBB, offering a potential solution for the treatment of diseases such as Alzheimer’s and brain tumors (Saraiva C. et al., 2016).

In therapeutic applications, NPs can act as anti-cancer agents by delivering cytotoxic drugs directly to tumor cells, thereby reducing the adverse effects associated with traditional chemotherapy. NP-based hyperthermia therapy, where magnetic NPs are exposed to an alternating magnetic field to generate heat, has been explored as a treatment for cancer, demonstrating the ability to selectively kill tumor cells (Laurent et al., 2011).

### 1.5 Scope and purpose of review

**FIGURE 2:**
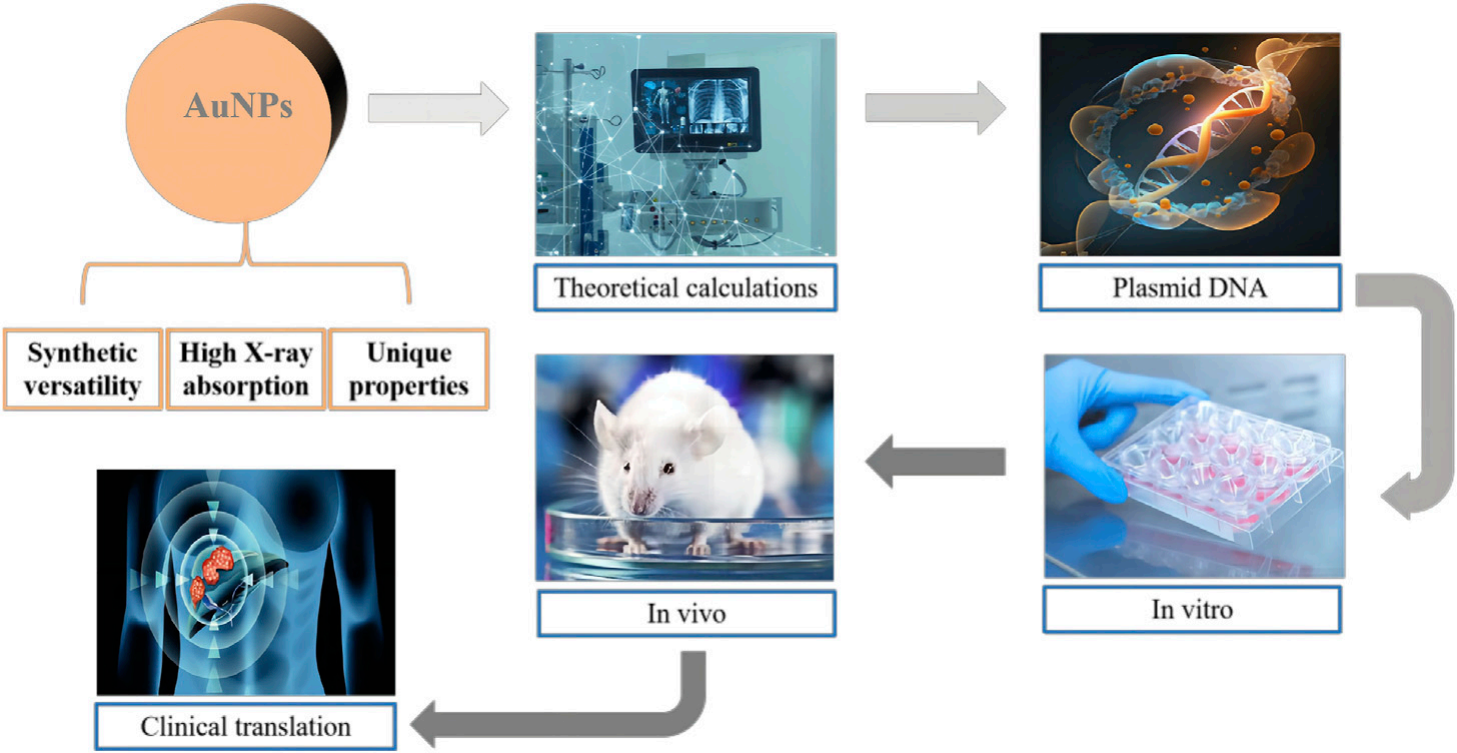
Schematic illustration of the potential of gold nanoparticles (AuNPs) as innovative radiosensitizers. The figure highlights the effective X-ray absorption, versatile synthesis, and distinctive chemical and optical properties of AuNPs. The interdisciplinary research efforts aimed at uncovering the mechanisms behind the enhanced radiation effects of AuNPs are also represented.

NERT represents a burgeoning field within oncological treatments, aiming to augment the efficacy of conventional radiotherapy through the integration of nanotechnology (Figure 2). The rationale for incorporating NPs lies in their unique physicochemical properties, which can be harnessed to enhance radiosensitization, improve tumor targeting, and reduce damage to surrounding healthy tissues (Hainfeld et al., 2004; Her et al., 2017).

NERT leverages the unique properties of NPs to enhance the efficacy of radiotherapy. The mechanisms of action involve physical dose enhancement, resulting from the increased absorption of radiation energy by high-Z materials, and biological/chemical radio sensitization, mediated by the modulation of cellular pathways and tumor microenvironment (McMahon et al., 2011; Butterworth et al., 2012).

Furthermore, the surface of NPs can be engineered to recognize and bind to specific tumor markers, allowing for selective accumulation within the tumor microenvironment (Dreaden et al., 2012). This targeted approach not only bolsters the therapeutic index of radiotherapy but also enables the use of NPs as diagnostic agents, facilitating image-guided radiotherapy (Kunjachan et al., 2015).

The scope of this review is to critically examine the current state of NERT, analyzing preclinical and clinical studies, and to elucidate the underlying mechanisms by which NPs potentiate radiotherapy. The review will also address the challenges faced in translating NERT from bench to bedside, including issues of biocompatibility, toxicity, and regulatory hurdles.

## 2 Enhanced application of NPs in brain tumor therapy

The advent of NP technology in brain tumor therapy marks a pivotal shift towards surmounting the formidable barriers posed by the brain’s protective mechanisms and the complex tumor microenvironment. This integrated section elucidates the multifaceted roles of NPs in drug delivery and treatment enhancement, underlining the strategic selection of NP types based on their unique attributes and therapeutic potential (Su et al., 2014).

### 2.1 Nanoparticle-driven strategies for targeted therapy

NPs, with their diverse compositions and customizable surfaces, present a novel paradigm for the targeted delivery of therapeutic agents to brain tumors. The strategic selection of NPs is predicated on their physicochemical properties, biocompatibility, and ability to navigate the blood-brain barrier (BBB), thereby ensuring precise delivery and controlled release of drugs within the tumor site. Key NP types demonstrating significant promise in preclinical models include:

Metallic NPs: Metallic NPs, encompassing gold, silver, and iron oxide, have garnered widespread application in brain tumor treatment due to their high surface area, optical, and magnetic properties. These NPs serve as effective drug carriers, facilitating efficient drug delivery through adsorption or encapsulation. Research indicates that silver NPs can suppress tumor cell proliferation and invasion, inhibiting tumor growth through the induction of cell apoptosis and cell cycle arrest. Furthermore, metallic NPs can achieve photothermal therapy by exploiting surface plasmon resonance, effectively eradicating tumor cells through localized hyperthermia (Huang et al., 2007; Zhang X. F. et al., 2016; Shi et al., 2017).

Polymeric NPs: Polymeric NPs, commonly employed as carriers, boast biocompatibility and tunable release capabilities. In brain tumor treatment, these NPs find widespread utility in delivering chemotherapy agents and genetic materials. For instance, poly (lactic-co-glycolic acid)-hydroxyapatite NPs efficiently transport chemotherapy drugs, enhancing therapeutic outcomes by controlling release rates. Moreover, surface modifications of polymeric NPs can enhance their efficacy in brain tumor treatment, achieved through selective drug release by targeting specific receptors on tumor cell surfaces (Agrawal et al., 2015).

Carbon-Based Nanomaterials: Emerging as a novel class of nanomaterials, carbon-based nanomaterials exhibit notable biocompatibility and safety. These materials have been extensively researched and applied in brain tumor treatment. Graphene nanosheets, characterized by their unique two-dimensional structure and high surface area, play a pivotal role in brain tumor treatment. Research demonstrates that graphene nanosheets inhibit tumor growth by inducing tumor cell apoptosis and cell cycle arrest, as well as enhance photothermal effects for photothermal therapy (Zhao et al., 2017).

Lipid NPs: LNPs are engineered to mimic cellular membranes, enhancing their compatibility with biological systems and facilitating seamless BBB penetration. Their capacity for controlled drug release positions them as effective carriers for chemotherapy agents and genetic material, directly targeting tumor cells while sparing healthy tissue (Ahmadi et al., 2020).

Silicon NPs: These NPs feature adjustable pore sizes, making them versatile carriers for a range of therapeutic agents. Their surface can be modified to bind specifically to tumor-associated markers, enabling targeted therapy and potentially reducing off-target effects (Mahawar et al., 2023).

Magnetic NPs: Leveraging the principle of magnetic guidance, these NPs can be directed to tumor sites with precision, offering applications in drug delivery and hyperthermia treatment. The localized heat generated by magnetic NPs under an external magnetic field can induce tumor cell death, adding a physical dimension to the therapeutic arsenal (Estrader et al., 2022).

Cell membrane-camouflaged NPs represent an innovative strategy for brain tumor treatment. By coating NPs with cell membranes derived from red blood cells, platelets, or tumor cells, these biomimetic NPs can evade immune clearance, cross the BBB, and specifically target tumor cells. For instance, Wang et al. developed a hypoxia-triggered RNAi nanomedicine camouflaged with glioblastoma cell membranes for synergistic chemo/radiotherapy (Wang et al., 2023). This biomimetic NP could efficiently deliver siRNA and chemotherapy drugs to glioblastoma cells, achieving hypoxia-activated gene silencing and drug release. In orthotopic glioblastoma mouse models, this nanomedicine significantly enhanced the efficacy of radiotherapy and prolonged survival, demonstrating the potential of cell membrane-camouflaged NPs for brain tumor treatment.

**FIGURE 3:**
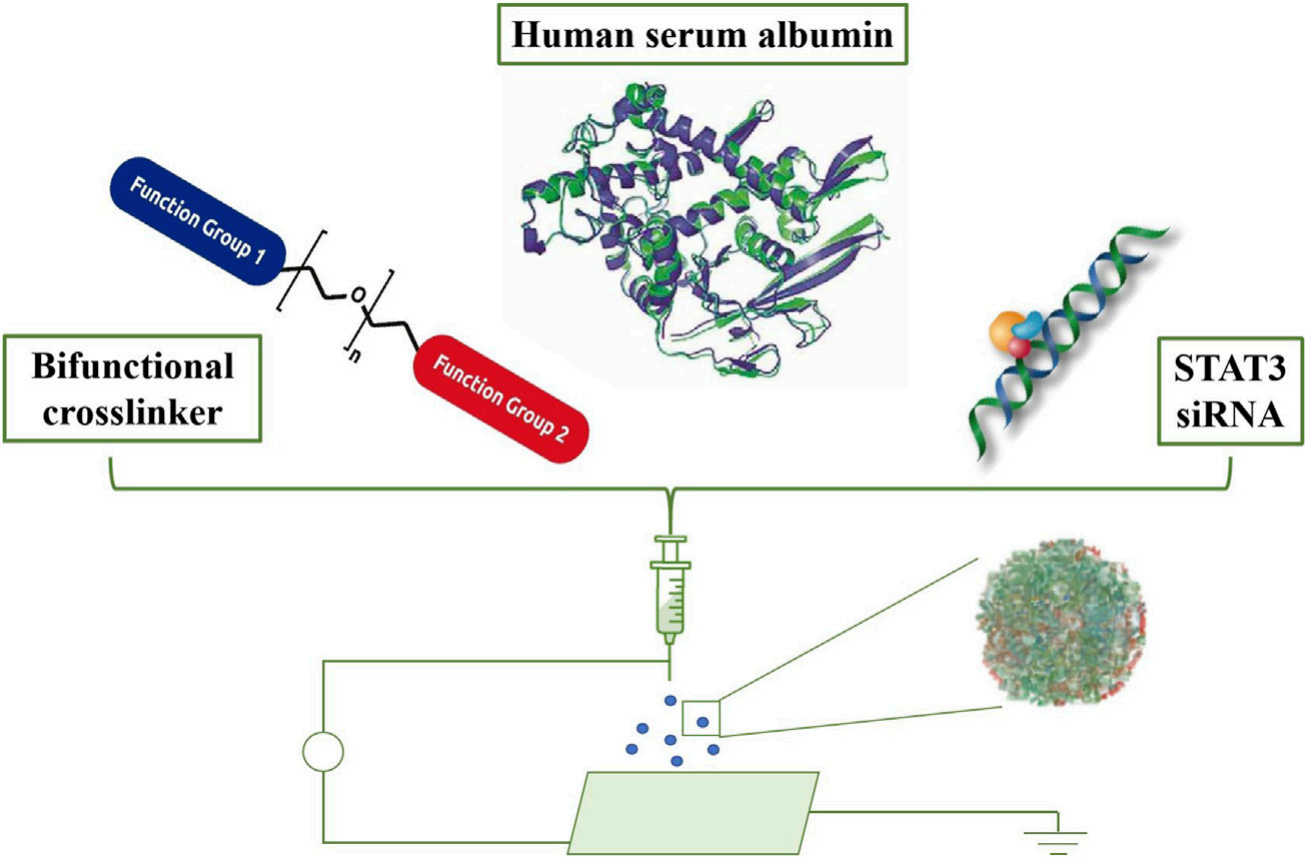
Formulation and *in vivo* biodistribution of targeted albumin nanoparticles. Schematic representation of the formulation process, including crosslinking, loading of STAT3 inhibitors, and iRGD conjugation. Adapted from Gregory et al., 2020.

Lastly, researchers have employed NPs for immunotherapy in brain tumor treatment. NPs activate the immune system, bolstering immune responses against brain tumors (Thakkar et al., 2010). Research reveals that encapsulating antigens and immune adjuvants within biological NPs achieves immunotherapy for brain tumors (Figure 3; Gregory et al., 2020).

### 2.2 Nanoparticle design for BBB penetration

The BBB is a highly selective permeability barrier that protects the brain from pathogens and controls the homeostasis of the central nervous system. However, this protective mechanism also restricts the entry of most therapeutic agents, posing a significant challenge in treating brain tumors. NPs have emerged as a versatile platform for drug delivery, offering the potential to bypass the BBB and target brain tumors effectively. The design of NPs, including their size, charge, and surface modifications, is crucial for enhancing their penetration through the BBB and ensuring their therapeutic efficacy.

#### 2.2.1 Size

Research has demonstrated that NPs within the size range of 20–100 nm exhibit optimal BBB penetration (Chen et al., 2022; Salatin et al., 2022). This size range leverages the EPR effect, allowing NPs to accumulate preferentially in tumor tissues due to the leaky vasculature characteristic of tumor microenvironments. Smaller NPs (<20 nm) may be rapidly cleared from the bloodstream, while larger NPs (>100 nm) may face difficulties in crossing the endothelial cell layer of the BBB. Therefore, designing NPs within this optimal size range is critical for maximizing their delivery to brain tumors.

#### 2.2.2 Charge

The surface charge of NPs significantly influences their interaction with the BBB. A neutral or slightly negative surface charge is preferred to minimize non-specific interactions with the BBB’s endothelial cells, which can lead to opsonization and clearance by the immune system (Simonis et al., 2022; Devaraj and Karthik Shree, 2023). Neutral or slightly negative NPs exhibit enhanced penetration through the BBB, likely due to reduced electrostatic repulsion with the negatively charged cell membranes. Consequently, careful control of NP surface charge is essential for improving BBB penetration and targeting efficiency.

#### 2.2.3 Surface and core functional groups

The functionalization of NPs with specific ligands targeting receptors overexpressed on the BBB or tumor cells is a promising strategy for enhancing BBB penetration and tumor targeting. Ligands such as transferrin or peptides can facilitate receptor-mediated transcytosis, allowing NPs to cross the BBB more efficiently (Seven et al., 2019; Simonneau et al., 2021; Choi et al., 2022). Additionally, the incorporation of polyethylene glycol (PEG) chains, a process known as PEGylation, can extend the circulation time of NPs by reducing their recognition and clearance by the immune system (Chen et al., 2022; Salatin et al., 2022). PEGylation also contributes to minimizing non-specific interactions with non-target cells, further enhancing the specificity and efficacy of NP-based therapies.

Several innovative strategies have been developed to facilitate NP penetration of the BBB for enhanced brain tumor treatment. Receptor-mediated transcytosis has shown promise, with studies demonstrating the effectiveness of targeting receptors such as transferrin (Baghirov, 2023), low-density lipoprotein, and nicotinic acetylcholine for NP transport across the BBB. Cell-penetrating peptides, such as TAT and penetration, have also been successfully employed to enhance NP translocation into the brain parenchyma (Thuenauer et al., 2017).

Temporary BBB disruption methods, including focused ultrasound (FUS) and osmotic agents, have shown promising results in preclinical studies (Gasca-Salas et al., 2021). FUS-mediated BBB opening has been used to enhance the delivery of various NP formulations, including liposomes and polymeric NPs, leading to improved tumor accumulation and therapeutic efficacy. Osmotic agents, such as mannitol, have also been employed to transiently disrupt the BBB and facilitate NP entry into the brain.

Comparative studies have provided valuable insights into the relative effectiveness of these strategies. For example, a study compared the BBB penetration and brain tumor accumulation of transferrin-functionalized gold NPs and TAT-conjugated gold NPs, demonstrating the superior performance of the transferrin-targeted NPs (Durant et al., 2018). Another study evaluated the efficacy of FUS-mediated BBB opening *versus* osmotic disruption for the delivery of polymeric NPs to glioblastoma, revealing a more localized and controlled NP accumulation with the FUS approach. These recent advancements highlight the potential of NERT strategies to overcome the BBB and enhance brain tumor treatment, with each approach offering unique advantages and considerations for clinical translation (Mehta et al., 2023).

## 3 Principles of radiotherapy and interactions with NPs

### 3.1 Categories of therapeutic radioactive particles

External Beam Radiotherapy (EBRT): EBRT is a common radiotherapy method that precisely irradiates tumor sites using external radiation beams. Various particles and energies, such as X-rays, γ-rays, and protons, can be employed to achieve targeted tumor treatment. Optimizing treatment plans maximizes tumor cell destruction while minimizing damage to normal tissues. Data-driven treatment planning has become a key strategy to enhance treatment outcomes (Langendijk et al., 2013; Malakhov et al., 2018; Nunna et al., 2021).

Brachytherapy: Also known as internal radiotherapy, brachytherapy involves placing radioactive isotopic sources near tumors for localized treatment. This approach delivers high radiation doses while minimizing damage to surrounding normal tissues. Advances in image-guided precise placement have improved brachytherapy outcomes for various cancers (Marinello et al., 1985; Potter et al., 2021).

Radioactive Isotope-Guided NP Therapy: This innovative radiotherapy strategy loads radioactive isotopes onto NPs, enabling targeted local tumor treatment through NP-guided delivery. This approach releases radioactivity within tumor cells, maximizing tumor tissue destruction while minimizing impact on surrounding tissues. This strategy combines nanotechnology and radiotherapy, offering new hope for brain tumor treatment (Giuliano et al., 2018).

Proton Therapy: Proton therapy, a precise radiotherapy method, employs high-energy proton beams to target tumors, achieving more accurate radiation treatment while reducing damage to surrounding normal tissues. Protons have a relatively high relative biological effectiveness, releasing more energy within tumor cells for improved treatment effects (Tsujii and Kamada, 2012; Paganetti, 2014).

αParticle Radiotherapy: α particle radiotherapy utilizes high-energy, highly ionizing α particles to directly damage tumor cells. Due to α particles’ high energy release, they inflict significant damage within a short range, presenting potential as a new avenue for radiotherapy (McDevitt et al., 2001; Henriksen et al., 2003).

### 3.2 Principles of radiotherapy and radiation biological effects

#### 3.2.1 Principles of radiotherapy

The goal of radiotherapy is to damage tumor cells by introducing high-energy radiation particles to inhibit their growth and division. These particles can be X-rays, γ-rays, protons, or heavy ions such as carbon ions (Table 1). Radiative particles interact with molecules within cells, triggering a series of biological effects that lead to cell damage and death. The objective of radiotherapy is to maximize damage to tumor cells while minimizing harm to surrounding normal tissues (Sorce et al., 2021).

**TABLE 1:** Enhanced classification and insights on radioactive particles in NERT.

Radioactive particles	Method introduction	Key advantages	Potential applications	Challenges in brain tumor radiotherapy	Adverse effects of brain radiotherapy	References
EBRT	EBRT precisely irradiates tumor sites using external radiation beams	Minimizes exposure to surrounding healthy tissues; adaptable to various tumor types	Widely used in treating brain, breast, prostate, and lung cancers	Limited penetration of radiosensitizers and chemotherapeutics due to the blood-brain barrier	Long-term neurocognitive impairment of normal brain tissues, especially in pediatric patients	Qu et al. (2020), Antonucci et al. (2022)
Brachytherapy	Delivers high radiation doses directly to the tumor site, minimizing damage to surrounding normal tissues	High precision; reduced systemic side effects; shorter treatment duration	Effective for prostate, cervical, and breast cancers	Difficulty in defining target volumes for diffusely infiltrative tumors like high-grade gliomas	Complications such as radiation-induced brain edema and radionecrosis	Di et al. (2022), Peng et al. (2022)
Radioactive Isotope-Guided NP Therapy	Utilizes NPs loaded with radioactive isotopes for targeted tumor treatment	Enhanced targeting capability; minimizes impact on surrounding healthy tissues	Emerging application in brain and pancreatic tumors	Restricted radiation dose to tumor areas due to limited tolerance of normal brain tissue	Other toxicities like alopecia and skin damage	Moravan et al. (2016)
αParticle Radiotherapy	Employs high-energy, highly ionizing α particles to directly damage tumor cells	Significant damage within a short range; high potential for hard-to-treat tumors	Investigational use in treating bone metastases and certain types of leukemia	Radio resistance of hypoxic tumor regions	Potential toxicity to surrounding normal brain tissues due to high LET	Qian et al. (2022)

#### 3.2.2 Radiation biological effects

When radiation interacts with biological tissues, it induces a range of radiation biological effects. These effects can be classified into two categories: direct effects and indirect effects.

Direct Effects: Radiation particles directly interact with DNA, proteins, and other molecules within cells, causing changes in molecular structures. This can result in DNA breaks, base damage, and cross-linking, disrupting normal biological functions of cells. Cells might fail to repair DNA damage, leading to cell death.

**FIGURE 4:**
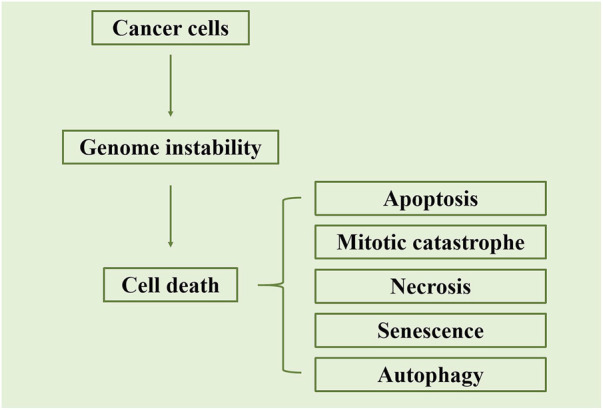
Schematic representation of the primary cell death mechanisms triggered by radiation. The figure illustrates that radiation-induced cell death occurs mainly through apoptosis or mitotic catastrophe. Apoptosis is characterized by the activation of caspases, leading to the formation of apoptotic bodies, while mitotic catastrophe results from aberrant mitosis and the formation of giant cells with multiple nuclei. Adapted from Baskar et al., 2012.

Indirect Effects: Radiation particles interact with water molecules within cells, generating free radicals and other highly reactive species. These free radicals can react with molecules within cells, triggering intracellular oxidative stress and damage. Indirect effects can impact normal cell metabolism and functions, ultimately leading to cell death (Figure 4; Baskar et al., 2012).

Recent research advancements have indicated that different types of radiation particles exhibit distinct mechanisms of action on tumor cells and normal cells. For instance, proton and heavy ion radiation particles, due to their high energy release, induce more DNA double-strand breaks within tumor cells, thereby causing more severe damage to them. Conversely, these particles, owing to their shallower penetration depth, have the potential to reduce harm to surrounding healthy tissues (Paganetti, 2014; Corvo et al., 2016; Grassi et al., 2019).

### 3.3 Mechanisms of interaction between NPs and radiation particles

The interaction between ionizing radiation and NPs is central to the efficacy of NERT in brain tumor treatment. High-Z material-based NPs, such as gold or gadolinium, can enhance the local dose deposition within the tumor through the increased generation of secondary electrons and reactive oxygen species. This physical dose enhancement effect is complemented by chemical radio sensitization, whereby NPs can be designed to deliver radio sensitizing agents specifically to the tumor site. Furthermore, the biological interactions between NPs and the tumor microenvironment, such as immune activation and vascular normalization, may contribute to the overall therapeutic response (Corvo et al., 2016).

It is worth noting that NPs can exhibit a phenomenon known as the radiation enhancement effect. When NPs interact with radiation, they may trigger local physical and chemical effects, increasing the deposition of radiation energy within cells. For instance, metal NPs can generate high-energy electrons under radiation exposure, which can damage surrounding DNA and cell membranes, further amplifying the effect of radiation therapy (Corvo et al., 2016). Additionally, NPs can produce a hyperthermic effect through interaction with radiation, causing localized heating and increasing cell sensitivity. Research has found that iron oxide NPs can enhance tumor cell sensitivity to X-rays, augmenting radiation-induced damage. This discovery provides new evidence for the application of NPs in radiation therapy (Hadi et al., 2019).

Furthermore, studies suggest that polymer NPs can enhance the sensitivity of brain tumor cells to radiation therapy, thereby improving treatment efficacy by intensifying the intracellular damage caused by radiation (Corvo et al., 2016). The radiation enhancement effect plays a crucial role in radiation particle therapy, positioning NPs as potential adjuvants in radiation therapy. Designed NPs carrying radiation sensitizers have been found to promote radiation-induced damage and elevate treatment efficacy in radiation therapy (Wu et al., 2020). However, it is important to note that factors such as NP size, shape, composition, *etc.*, can influence their interaction with radiation. Thus, in NP-guided radiation particle therapy, a thorough investigation of these interaction mechanisms is required to achieve optimal treatment outcomes.

While physical dose enhancement, chemical radio sensitization, and biological interactions contribute to the efficacy of NERT, it is essential to acknowledge the limitations and potential drawbacks associated with each mechanism. For instance, the physical dose enhancement effect may be limited by the concentration and distribution of NPs within the tumor, as well as the energy of the radiation source. Chemical radio sensitization, although promising, may be hindered by the stability and specificity of the radio sensitizing agents. Biological interactions, such as immune activation, require further investigation to fully understand their implications in the context of NERT.

## 4 Advantages of NP-Guided radiation particle therapy

### 4.1 Targeted delivery advantage of NPs in brain tumor treatment

NPs offer significant advantages in targeted delivery for brain tumor treatment, particularly in the context of radiation particle therapy. Conventional radiation therapy often struggles to avoid harming surrounding normal brain tissues, leading to severe neurological dysfunction and cognitive impairment. However, NPs, acting as carriers, enable highly targeted delivery of drugs and radioactive isotopes to tumor sites, thereby minimizing exposure of normal tissues (Chang et al., 2019).

Through the action of NPs, radioactive isotopes can be precisely guided to areas adjacent to tumor cells, facilitating localized radiation therapy. This targeted delivery advantage substantially reduces radiation exposure to surrounding normal brain tissues, lowering the risks of neurological dysfunction and cognitive impairment. Furthermore, by adjusting their surface properties, NPs can more readily bind specifically to tumor cells, further enhancing treatment precision. A study by Li et al. demonstrated that NPs can traverse the BBB to achieve targeted delivery to brain tumors, exhibiting excellent biocompatibility and drug-release capabilities. This finding offers novel avenues and methods for brain tumor treatment (Li J. et al., 2020). Wagner et al. discovered through experiments that NPs, by means of passive and active targeting mechanisms, can achieve accurate drug delivery within brain tumors, providing a more precise and effective approach to treatment (Wagner et al., 2012). Chen et al. found that NPs can facilitate gene therapy for brain tumors through gene delivery mechanisms, introducing a new personalized treatment strategy (Chen et al., 2017). Additionally, Yu et al. (2022) uncovered that NPs, besides achieving precise targeting, can enhance brain tumor treatment efficacy by reducing drug resistance. These findings offer innovative solutions for brain tumor treatment. Through BBB penetration, NPs achieve targeted delivery to brain tumors with good biocompatibility and drug-release capabilities. Moreover, NPs can elevate treatment efficacy through mechanisms such as photothermal effects, gene delivery, and reduction of drug resistance. These discoveries provide new insights and methodologies for brain tumor treatment. However, these studies have certain limitations. For instance, issues regarding the toxicity and long-term safety of NPs necessitate further research. Additionally, NP fabrication and application techniques require ongoing improvement and optimization. Future research should further explore the potential applications of NPs in brain tumor treatment and address the challenges faced by current studies.

### 4.2 Biological effects of NPs

NP-guided radiation particle therapy not only holds advantages in targeted delivery but also enhances treatment efficacy through its unique biological effects (Table 2).

**TABLE 2:** Comprehensive overview of biological effects of NPs in NERT.

Biological effect	Mechanism	Impact on cancer therapy	Insights	Key studies
Cellular Toxicity and Apoptosis	NPs can induce cellular toxicity and apoptosis through oxidative stress, cell membrane disruption, and DNA damage	Enhances the efficacy of radiotherapy by promoting tumor cell death	Studies suggest that the surface properties and cellular uptake of NPs, such as silver NPs, play a crucial role in inducing apoptosis	Gurunathan et al. (2018)
Autophagy	NPs influence the autophagy pathway, impacting cell survival and death	Can be leveraged to induce autophagic cell death in tumor cells, complementing radiotherapy	Research indicates that NPs, including titanium dioxide, can induce autophagy-mediated apoptotic cell death, offering a potential therapeutic strategy	Li et al. (2020b)
Oxidative Stress	NPs can lead to an imbalance in cellular redox states, resulting in oxidative stress	Oxidative stress enhances the generation of ROS, contributing to DNA damage and tumor cell kill	Gold NPs have been shown to enhance oxidative stress in chronic kidney disease cell models, indicating their potential in radio sensitization	Chen et al. (2022), Zhao et al. (2021)
Inflammation	Certain NPs can trigger inflammatory responses by activating the NLRP-3 inflammasome	While inflammation can contribute to tumor progression, controlled use of NPs can potentially enhance the immune response against tumors	Silver NPs have been found to induce degradation of the endoplasmic reticulum stress sensor, leading to activation of the NLRP-3 inflammasome	(Simard et al., 2015)

### 4.3 Cellular toxicity and apoptosis

The cellular toxicity and apoptosis induced by NPs are important considerations for researchers. Studies suggest that NPs can induce cellular toxicity and apoptosis through various pathways, including oxidative stress, cell membrane disruption, DNA damage, *etc.* For instance, research indicates that silver NPs can induce apoptosis and mitochondrial damage in human lung epithelial cells, an effect possibly linked to the surface properties and cellular uptake of these NPs (Gurunathan et al., 2018). Moreover, some studies have found that factors such as NP size, shape, and surface modifications can also impact their cellular toxicity and apoptosis effects (García-Torra et al., 2021).

### 4.4 Autophagy

Autophagy is a cellular self-degradation process that provides nutrients and energy by engulfing cellular proteins and organelles. Recent research indicates that NPs can influence the autophagy pathway, thereby impacting cell survival and death. For example, a study revealed that titanium dioxide NPs can suppress the autophagy pathway, leading to cell death (Li J. et al., 2020). Another study suggested that NPs of iron oxide can mitigate cell damage induced by oxidative stress by promoting autophagic degradation.

### 4.5 Oxidative stress

Oxidative stress refers to a series of reactions resulting from the imbalance in cellular redox equilibrium, potentially leading to oxidative damage of proteins, lipids, and DNA. NPs can induce oxidative stress through various means, including direct interaction with intracellular molecules and induction of free radical production. Recent research suggests that the oxidative stress effects of NPs may be linked to their surface chemistry and biodegradability. For instance, a study found that the oxidative stress effects of NPs of iron oxide are related to their surface modifications, with different modifications possibly leading to varying degrees of oxidative stress responses (Xuan et al., 2023).

### 4.6 Cellular signaling pathway regulation

NPs can regulate cellular signaling pathways through multiple avenues, including activating or inhibiting specific pathways and influencing cytokine production. Recent research indicates that the signaling pathway regulation effects of NPs may be associated with their surface properties and cellular uptake. For instance, a study found that titanium dioxide NPs can induce inflammation by activating the Toll-like receptor four signaling pathway. Another study suggested that gold NPs can modulate immune responses by affecting cytokine production (Swartzwelter et al., 2020).

### 4.7 Immune activation

NPs can activate immune responses through various means, including inducing cytokine production and eliciting inflammation. Recent research suggests that the immune activation effects of NPs may be linked to their surface properties and cellular uptake. For example, a study found that silver NPs can induce inflammation by activating the Toll-like receptor two signaling pathway (Kim et al., 2012). Another study indicated that silica NPs can activate immune responses by inducing cytokine production (Nakanishi et al., 2016).

### 4.8 Gene and protein expression regulation

NPs can regulate gene and protein expression through various means, including influencing transcription factor activity and modulating epigenetic modifications. Recent research suggests that the effects of NPs on gene and protein expression regulation may be associated with their surface properties and cellular uptake. For instance, a study found that titanium dioxide NPs can affect gene expression by modulating transcription factor activity (Alijagic et al., 2019). Another study indicated that zinc oxide NPs can impact protein expression by regulating epigenetic modifications (Safar et al., 2019).

In conclusion, the biological effects of NPs are a complex issue encompassing multiple areas of research. Recent studies suggest that the biological effects of NPs may be closely related to factors such as their surface properties, size, shape, and biodegradability. Further research will contribute to a comprehensive understanding of the biological effects of NPs and provide guidance for their applications in the fields of medicine and biology (Youden et al., 2022).

## 5 Review of clinical trials and research applications

### 5.1 NERT in specific brain tumor types and subtypes

NERT has been investigated in various brain tumor types and subtypes, each presenting unique challenges and opportunities for clinical translation. In GBM, gold nanoparticles (AuNPs) have been the most extensively studied, with preclinical studies demonstrating enhanced radio sensitization and improved survival in animal models. However, the heterogeneity and infiltrative nature of GBM pose significant challenges for NP delivery and distribution within the tumor (Peng et al., 2020). Strategies to overcome these barriers include the use of tumor-targeting ligands and convection-enhanced delivery (Georgiou et al., 2023).

In meningiomas, AuNPs have shown promise in enhancing the efficacy of radiation therapy, particularly in atypical and malignant subtypes (Khoobchandani et al., 2021). However, the relatively low incidence of high-grade meningiomas has limited the clinical translation of NERT in this setting. Future studies should focus on identifying biomarkers to predict which meningiomas are most likely to benefit from NERT (Luo et al., 2020).

For PNETs, such as medulloblastoma, NERT has the potential to improve treatment outcomes while minimizing the long-term neurocognitive sequelae associated with conventional radiotherapy (Treisman et al., 2022). Preclinical studies using AuNPs and iron oxide NPs have shown encouraging results in medulloblastoma models. However, the developing brain presents unique challenges for NP delivery and toxicity assessment. Future research should prioritize the development of biocompatible and targeted NP formulations for pediatric brain tumors (Edelstein et al., 2011).

In the context of brain metastases, NERT has the potential to improve local control and reduce the need for whole-brain radiation therapy, which is associated with significant neurocognitive decline. Preclinical studies using AuNPs have demonstrated enhanced radio sensitization in brain metastasis models (Rosoff et al., 2022). However, the heterogeneity of primary tumor types and the presence of multiple metastatic lesions complicate the clinical translation of NERT in this setting. Future studies should investigate the use of NERT in combination with systemic therapies and immunotherapy to address both local and distant disease control (Rosoff et al., 2022).

In summary, NERT has shown promise in various brain tumor types and subtypes, but each presents unique challenges and considerations for clinical translation. Future research should focus on developing targeted and biocompatible NP formulations, optimizing delivery strategies, and investigating combination approaches to maximize the therapeutic potential of NERT in specific brain tumor contexts.

### 5.2 Efficacy evaluation

NP-enhanced radiotherapy, as a novel treatment strategy, has gained significant attention in clinical trials and research. Over the past few years, researchers have conducted a series of clinical studies to evaluate its application in patients with brain tumors. These studies involve NP selection, guided radioisotope delivery, optimization of treatment plans, as well as safety and efficacy assessment (Youden et al., 2022).

**FIGURE 5:**
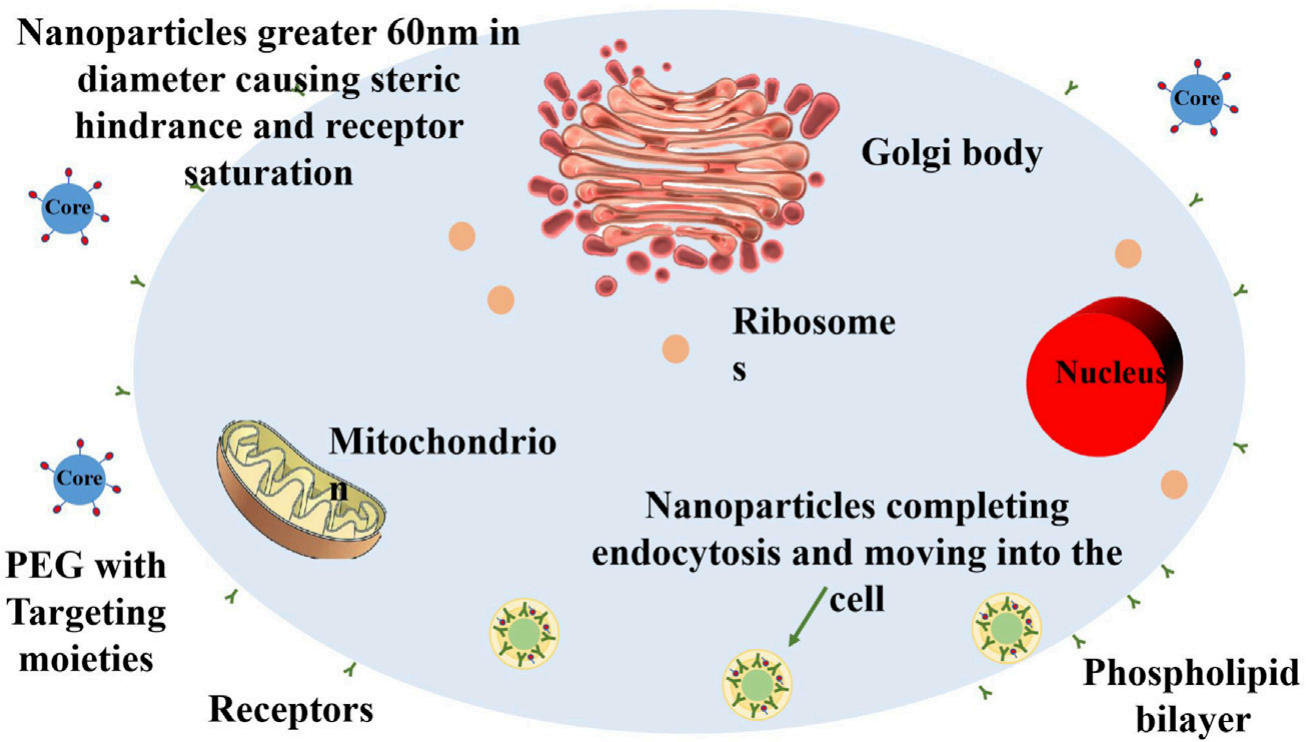
Schematic illustration of the size-dependent membrane-wrapping behavior of nanoparticles (NPs). The figure shows that NPs with diameters above 60 nm drive the membrane-wrapping process by binding extensively to receptors, while NPs below 30 nm attach to the membrane but require proximity for efficacy. NPs with diameters between 30 and 60 nm effectively drive membrane-wrapping. Redrawn based on the content of Hoshyar et al., 2016.

In terms of NP selection, metal NPs and polymer NPs have been particularly studied. Metal NPs exhibit radiation-enhancing effects and local hyperthermia, giving them unique advantages in radiotherapy. Polymer NPs, on the other hand, possess excellent drug carrier properties and can facilitate combined delivery of chemotherapy agents and radioisotopes for multi-modal therapy. Different types of NPs play distinct roles in treatment strategies, providing patients with more therapeutic options (Figure 5; Hoshyar et al., 2016).

### 5.3 Preclinical insights

The clinical application of NP-enhanced radiotherapy has shown promising preliminary results in terms of safety and efficacy assessment. Researchers have monitored treatment processes, side effects, and treatment outcomes in brain tumor patients. Initial findings indicate that NP-guided radiotherapy presents significant advantages in reducing damage to normal brain tissue compared to traditional radiotherapy. Patients’ neurological function and quality of life have been preserved to some extent, positively impacting their recovery and wellbeing (Zhang Y. N. et al., 2016).

Preclinical research has been pivotal in unveiling the potential of NERT for the treatment of brain tumors. Furthermore, NP-guided radiotherapy might enhance treatment outcomes through biological effects. Local hyperthermia can enhance the effects of radiotherapy, making tumor cells more susceptible to damage. Immune activation effects can stimulate the immune system’s attack on tumors, increasing treatment durability. These effects are gradually becoming evident in clinical applications, providing positive indications for the future development of NP-enhanced radiotherapy strategies (Bag et al., 2023). By selecting appropriate NPs, optimizing treatment plans, and evaluating safety and efficacy, this strategy offers new therapeutic opportunities for brain tumor patients, bringing new hopes for improved treatment outcomes and prognosis (Table 3).

**TABLE 3:** Nanoparticles developed for radiotherapy in brain tumor treatment.

Nanoparticle type	Key properties	Applications in RT	Therapeutic benefits	References
Metallic NPs	High surface area, optical and magnetic properties	Drug delivery, Photothermal therapy	Efficient drug delivery, tumor cell eradication through localized hyperthermia	Huang et al. (2007), Zhang et al. (2016a), Shi et al. (2017)
Polymeric NPs	Biocompatibility, tunable release capabilities	Delivery of chemotherapy agents and genetic materials	Enhanced therapeutic outcomes, selective drug release targeting tumor cells	Agrawal et al. (2015)
Carbon-Based Nanomaterials	Biocompatibility, high surface area	Photothermal therapy	Inhibition of tumor growth, induction of tumor cell apoptosis and cell cycle arrest	Zhao et al. (2017)
Lipid NPs	Biocompatible, BBB penetration	Drug and genetic material delivery	Precise targeting, controlled release, reduced systemic toxicity	Ahmadi et al. (2020)
Silicon NPs	Adjustable pore sizes, surface modifiability	Efficient drug carriers, targeted therapy	Enhanced drug delivery, reduced off-target effects	Mahawar et al. (2023)
Magnetic NPs	Responsive to external magnetic fields, induces hyperthermia	Directed drug delivery, hyperthermia treatment	Targeted therapy, localized tumor cell destruction	Estrader et al. (2022)

## 6 Challenges and future directions

The comparative analysis of NERT strategies for BBB penetration has provided valuable insights into their relative effectiveness and potential for clinical translation. However, several challenges remain in optimizing these approaches for targeted delivery and specificity. The heterogeneity of brain tumors and the complexity of the BBB microenvironment necessitate the development of more sophisticated NP designs that can adapt to these variations (Kim and Lee, 2022).

Future research should focus on the systematic evaluation of different BBB penetration strategies across a range of brain tumor models, considering factors such as NP size, surface chemistry, and targeting ligand density (Reddy et al., 2021). The integration of multiple strategies, such as combining receptor targeting with temporary BBB disruption, may offer synergistic effects and warrants further investigation. Additionally, the long-term safety and neurocognitive impact of these approaches must be carefully assessed in relevant preclinical models and clinical trials.

Addressing these challenges and advancing the comparative analysis of NERT strategies for BBB penetration will be crucial for the successful translation of these innovative approaches into clinical practice, ultimately improving the outcomes for patients with brain tumors.

### 6.1 Targeted delivery and specificity

Targeting NPs to brain tumors involves both passive and active mechanisms. Passive targeting exploits the enhanced permeability and retention (EPR) effect, while active targeting uses ligands to bind specific receptors on tumor cells (Jain, 2012; Saraiva J. et al., 2016). Advanced *in vivo* models and clinical trials are essential to evaluate the efficacy and safety of these targeted NP systems.

### 6.2 Optimization of NP formulations

Optimizing NP formulations for radio sensitization is crucial for treating radioresistant tumors. High-Z material-based NPs, such as gold, gadolinium, and hafnium, can significantly increase radiation absorption and DNA damage. Surface modification with radio sensitizing drugs or molecules that enhance tumor oxygenation can further amplify therapeutic outcomes (Hainfeld et al., 2014; Kunjachan et al., 2015).

The systemic toxicity of NPs is a significant concern that limits their clinical translation. Optimization strategies are focused on improving the biocompatibility and reducing the off-target effects of NPs. Surface engineering of NPs with polyethylene glycol has been widely adopted to enhance their circulation time and reduce opsonization, thereby diminishing mononuclear phagocyte system uptake and minimizing toxicity (Knop et al., 2010). Additionally, the development of biodegradable NPs that can safely disassemble and be cleared from the body after fulfilling their therapeutic purpose is gaining traction (Park et al., 2011).

The design of NPs with intrinsic antioxidant properties or surface functionalization with anti-inflammatory agents can also mitigate oxidative stress and inflammation associated with NP administration (Sengupta et al., 2018). Moreover, the use of *in silico* modeling and high-throughput screening can predict NP toxicity and optimize their formulations before *in vivo* application. The optimization of NP formulations for enhanced radio sensitization and reduced toxicity is a crucial avenue for future research. However, the development of such formulations may be complicated by the complex interplay between NP properties, tumor microenvironment, and radiation response.

### 6.3 Personalization of therapy

Personalized nanomedicine is an emerging paradigm that aims to tailor NP-based therapies to the individual characteristics of a patient’s tumor. This approach necessitates the integration of comprehensive diagnostic information, including genetic, proteomic, and metabolic profiles, to design NPs that can selectively target and treat cancerous cells based on their unique molecular signatures (Schroeder et al., 1998).

The functionalization of NPs with specific ligands that recognize tumor-specific markers has shown potential in enhancing the selectivity and efficacy of cancer therapies. Recent advancements in the field have seen the development of multifunctional NPs capable of simultaneous imaging and therapy, allowing for real-time monitoring of treatment response and the adjustment of therapeutic regimens accordingly (Davis et al., 2013). The ongoing research and development in this field are likely to yield novel NP-based therapeutics that can overcome current limitations in cancer treatment and offer a more targeted, effective, and safer approach to cancer management. Personalization of NERT also presents significant challenges, as it requires a comprehensive understanding of the patient’s tumor biology and the ability to tailor NP design accordingly.

### 6.4 Integration with other therapies

#### 6.4.1 Combination with chemotherapy, immunotherapy, or targeted therapy

The integration of NP systems with conventional cancer therapies such as chemotherapy, immunotherapy, and targeted therapy is a burgeoning area of research that aims to enhance therapeutic efficacy while minimizing adverse side effects. This integrative approach leverages the unique properties of NPs to improve drug delivery, modulate the immune response, and facilitate targeted drug action.

Chemotherapy, one of the mainstays of cancer treatment, often suffers from poor specificity and systemic toxicity. NPs can serve as carriers for chemotherapeutic agents, enhancing their accumulation in tumor tissues through the EPR effect. For instance, the development of doxorubicin-loaded liposomal NPs has shown improved therapeutic outcomes by reducing cardiotoxicity and increasing tumor drug concentration (Barenholz, 2012). Similarly, polymeric NPs have been engineered to release chemotherapeutic agents in response to specific stimuli within the tumor microenvironment, thereby enhancing the precision of drug delivery (Wang et al., 2019).

Immunotherapy, which harnesses the patient’s immune system to combat cancer, can also benefit from NP integration. NPs can be designed to deliver immunomodulatory agents directly to the tumor site or lymphoid tissues, thus amplifying the immune response against cancer cells. For example, NPs loaded with checkpoint inhibitors have been reported to enhance antitumor immunity and show synergistic effects when combined with other therapeutic modalities (Patel et al., 2020).

Targeted therapy, which involves agents that specifically target molecular pathways essential for tumor growth and survival, has also seen advancements through NP integration. The functionalization of NPs with ligands that recognize tumor-specific antigens or receptors enables the selective delivery of targeted therapies, thereby reducing off-target effects. A notable example is the development of NPs conjugated with antibodies against the epidermal growth factor receptor (EGFR), which have shown enhanced targeting and treatment of EGFR-overexpressing tumors (Wang et al., 2007).

### 6.5 Synergistic effects and treatment protocols

The synergy between NPs and other cancer therapies can lead to the development of novel treatment protocols that offer superior efficacy over single-modality treatments. The combination of NPs with radiation therapy, as discussed previously, can be further enhanced by integrating chemotherapy or immunotherapy, leading to multimodal treatment regimens that exploit the strengths of each approach.

For instance, the simultaneous delivery of radio sensitizing NPs and DNA-damaging chemotherapeutic agents can lead to enhanced tumor cell kill due to the increased generation of reactive oxygen species and DNA damage. The sequential delivery of radiation therapy and NP-mediated chemotherapy has been shown to result in a synergistic tumor response, as radiation can increase the permeability of tumor vessels, thereby enhancing NP accumulation (Chen Q. et al., 2023).

Moreover, the combination of NPs with immunotherapy can result in synergistic effects by modulating the tumor microenvironment to be more conducive to immune cell infiltration and activity. NPs can be engineered to release cytokines or other immune-stimulating agents in response to radiation, thus potentiating the immune-mediated eradication of cancer cells (Duan et al., 2021).

### 6.6 Regulatory and manufacturing considerations for NERT

The production of NPs for NERT requires rigorous standardization to ensure safety, efficacy, and reproducibility. Key characteristics such as size, shape, surface charge, and drug loading efficiency must be controlled. Implementing Good Manufacturing Practice guidelines is vital for quality control and minimizing risks like contamination or dosage inconsistencies (Wilhelm et al., 2011; Rai et al., 2016).

The clinical translation of NERT faces several challenges, including the optimization of NP formulations for targeted delivery and radio sensitization, while minimizing toxicity (Tinkle et al., 2014). Navigating the regulatory landscape and addressing safety concerns will be critical for the successful implementation of NERT in clinical practice (Moghimi et al., 2012).

Navigating the regulatory landscape will be a critical hurdle in the clinical translation of NERT, necessitating close collaboration between researchers, manufacturers, and regulatory bodies to ensure the safety and efficacy of these novel treatments (Tinkle et al., 2014).

### 6.7 Potential toxicity of nanoparticles in humans

The increasing use of NPs in medical applications, including NER), has raised concerns about their potential toxicity in humans. While the unique properties of NPs make them attractive for therapeutic purposes, their small size and high surface area-to-volume ratio can also lead to unintended biological interactions and adverse effects (Lewinski et al., 2008).

One of the main concerns is the systemic toxicity of NPs following their administration. NPs can distribute to various organs and tissues, leading to accumulation and potential damage. The liver, spleen, and kidneys are particularly susceptible to NP toxicity due to their role in NP clearance and metabolism (Zhang Y. N. et al., 2016). *In vitro* and *in vivo* studies have shown that certain NPs can induce oxidative stress, inflammation, and DNA damage, which may contribute to long-term health consequences (Wang et al., 2013).

Another concern is the potential immunogenicity of NPs. Some NPs have been shown to activate the immune system, leading to the production of pro-inflammatory cytokines and the recruitment of immune cells (Yang et al., 2021). This immune activation can be beneficial in the context of cancer immunotherapy but may also lead to undesirable side effects and autoimmune reactions.

The long-term effects of NP exposure are still not fully understood. There is a need for more comprehensive toxicological studies to evaluate the chronic toxicity of NPs, including their potential carcinogenicity and genotoxicity (Fattal et al., 2014). Additionally, the environmental impact of NPs and their potential to accumulate in the food chain should be considered.

To mitigate the potential toxicity of NPs, several strategies have been proposed. These include the use of biocompatible and biodegradable materials, surface modification to reduce immunogenicity, and the development of targeted delivery systems to minimize off-target effects (Yang et al., 2021). Furthermore, rigorous safety assessment and regulatory guidelines are essential to ensure the safe and responsible use of NPs in medical applications. While the potential of NPs in NERT is promising, it is crucial to carefully consider and address their potential toxicity in humans. Ongoing research and safety evaluations are necessary to develop NPs that are both effective and safe for clinical use. By understanding and mitigating the potential risks, we can harness the full potential of NPs in the treatment of brain tumors and other medical applications.

## 7 Conclusion

NERT is an emerging approach in treating brain tumors, offering potential where conventional therapies are limited by the brain’s protective barriers and the sensitivity of surrounding tissues. The design of NPs for NERT involves careful consideration of size, surface charge, and functionalization to facilitate BBB crossing and targeted tumor accumulation. NPs in the size range of 20–100 nm, with neutral or slightly negative surface charge, and functionalized with targeting ligands such as transferrin or peptides, have shown promise in traversing the BBB and delivering therapeutic agents to brain tumors.

The use of NERT leverages the enhanced permeability and retention (EPR) effect for selective tumor accumulation, amplifying treatment effects while minimizing damage to healthy cells. Metallic NPs, such as gold and gadolinium, have demonstrated significant potential in enhancing radiation-induced damage, leading to improved tumor control and survival benefits in preclinical models.

However, clinical adoption of NERT is still in its early stages, focusing on safety, dosage, and NP distribution. Challenges remain, including tumor heterogeneity, consistency in NP delivery across the BBB, and long-term safety considerations. Additionally, the complex regulatory landscape and the need for standardization in NP production and characterization present hurdles in translating NERT from research to clinical practice.

Despite these challenges, NERT holds significant promise in improving outcomes for patients with brain tumors, particularly those with inoperable or treatment-resistant tumors, by enabling more precise and potent radiation delivery. The approach also opens doors to theranostic applications, combining treatment with diagnostic imaging for real-time monitoring and treatment adaptation.

Future directions in NERT for brain tumors include the development of multifunctional NPs that combine imaging, targeting, and therapeutic capabilities, as well as the integration of artificial intelligence and personalized medicine approaches to optimize treatment planning and delivery. Collaborative efforts among researchers, clinicians, and regulatory bodies will be crucial in addressing the challenges and realizing the full potential of NERT in the management of brain tumors.
